# Appropriate Endotracheal Tube Position for Percutaneous Dilatational Tracheostomy: A Single-Center Observational Study

**DOI:** 10.7759/cureus.51895

**Published:** 2024-01-08

**Authors:** Takahiro Michishita, Naoya Suzuki, Takeru Abe, Kento Nakajima, Masayasu Gakumazawa, Tomoki Doi, Ichiro Takeuchi

**Affiliations:** 1 Department of Emergency Medicine, Yokosuka Kyosai Hospital, Yokosuka, JPN; 2 Department of Emergency Medicine, Graduate School of Medicine, Yokohama City University, Yokohama, JPN

**Keywords:** surgical tracheostomy, prediction equation, percutaneous dilatational tracheostomy, endotracheal tube position, endotracheal tube

## Abstract

Aim

This study aimed to investigate the appropriate endotracheal tube (ETT) position during percutaneous dilatational tracheostomy (PDT).

Methods

This single-center observational study included hospitalized patients who underwent surgical tracheostomy (ST) between August 2021 and October 2022. During ST, the trachea was opened, and the ETT was pulled out visually. It stopped when the ETT was no longer visible, and the tracheostomy tube was placed in the trachea. The ETT position was measured by considering the ETT position during ST to be the appropriate position during PDT. The correlation between the measured ETT position and patient characteristics was evaluated. A prediction equation for the ETT position was derived from the derivation group, and validation of the prediction equation was evaluated by the validation group.

Results

Forty-six and 15 patients were in the derivation and validation groups, respectively. Weight, duration of intubation, and in-hospital mortality were significantly different between the two groups. The measured ETT position correlated with body height (r=0.60, p<0.001) and sex (r=0.45, p=0.002), while the ETT position before ST showed a weak correlation (r=0.34, p=0.020). The predicted and measured values in the validation group correlated with each other (r=0.58, p=0.024).

Conclusion

The appropriate ETT position for PDT correlates with body height, and the equation "body height×0.112-0.323 cm" was derived. This predictive equation may be useful as a guide for ETT positioning during PDT puncture.

## Introduction

Tracheostomy is one of the most frequently performed surgical procedures in the intensive care unit (ICU) on patients in whom extubation is difficult or who have been intubated for a long time [1-3]. There are two types of tracheostomies: surgical tracheostomy (ST) and percutaneous dilatational tracheostomy (PDT). ST was first performed in 1909 by surgically peeling the skin down to the anterior surface of the first or second tracheal cartilage rings, opening the trachea, and inserting a tracheostomy tube under direct vision [4]. PDT was first described in 1985, and although there are various methods for PDT, the modified Ciaglia method developed in 1998, in which a single dilator is used to expand the wound in a single step, is the most frequently used [5,6]. The prevalence of PDT varies regionally, with PDT accounting for 86% of tracheostomies performed in Germany and 97% in the United Kingdom; in France, PDT represents only 27% of all the tracheostomies performed [7-9]. PDT is preferred over ST throughout Europe because it requires less operative time and has a similar risk of surgery-related complications compared to ST; PDT is the first choice for tracheostomy in many countries [6,10,11].

PDT is commonly performed by puncturing the first or second tracheal cartilage, dilating, and inserting a tracheostomy tube while monitoring each step of PDT with a bronchoscope [1]. Bronchoscopy is used in 69.2% of all PDT procedures [6]. During the puncture, the endotracheal tube (ETT) position should be adjusted so that the puncture position and the ETT do not overlap. Although a method of puncturing the anterior surface of the trachea with the entire ETT without daring to move the ETT to protect the posterior tracheal wall has been reported [12], this method of adjusting the ETT position and puncturing is commonly used.

If the ETT position is deep during PDT, the ETT will be present under the puncture needle, leading to surgical errors and accidental puncture of the ETT and bronchoscope. Conversely, a shallow ETT position increases the risk of accidental extubation during tracheostomy. Accidental puncture of the ETT is the second most common complication of PDT after hemorrhage [6] and accidental extubation during surgery; breakage of the ETT cuff has also been reported [6,13,14]. There are hybrid methods of PDT and ST in which the subcutaneous tissue is removed from the front of the trachea, and the ETT is pulled out using the light source of the bronchoscope [15]. The hybrid method requires more time to remove the subcutaneous tissue to the front of the trachea, reducing the advantage of the shorter operative time of PDT [10]. Knowing the appropriate ETT position during PDT may lead to safer and shorter PDT. However, an appropriate ETT position and how much ETT position should be adjusted for PDT puncture have not been reported yet. This study aimed to investigate the appropriate ETT position for PDT using ST.

## Materials and methods

Research design

This was a retrospective single-center observational study conducted at Yokosuka Kyosai Hospital in Yokosuka, Japan, from August 2021 to October 2022 (approval number: 21-47). Patients who underwent ST in the emergency department were included in the study. The derivation group was studied from August 2021 to May 2022 and the validation group from June 2022 to October 2022. The ETT position was measured in the median of the portal teeth when ST was performed. The measured ETT position was used as the outcome. Data on age (years), sex (male or female), body height (cm), weight (kg), length of hospital stay (days), length of ICU stay (days), length of intubation (days), Sequential Organ Failure Assessment (SOFA) score at admission, Acute Physiologic Assessment and Chronic Health Evaluation (APACHE) II score at admission, in-hospital death, ETT tip position from the tracheal bifurcation on chest radiographs (cm), ETT position before ST (cm), diseases leading to tracheostomy, and tracheostomy complications were also collected from the electronic medical records. A prediction equation for ETT position was derived in the derivation group, and the prediction equation was validated using the validation group.

ETT position measurement method

**Figure 1:**
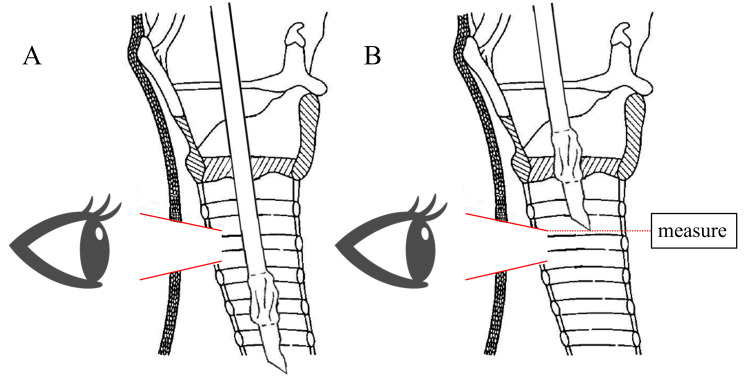
Method of ETT position measurement during ST A: The subcutaneous skin is peeled off and the trachea is opened. B: The ETT is pulled out such that the tip of the tube is no longer visible. From the Ciaglia Blue Rhino IFU images, Cook Medical. Copyright © Cook Medical. Reprinted and partially modified with permission from Cook Medical. ETT: endotracheal tube; ST: surgical tracheostomy

During ST, the subcutaneous skin was peeled, the trachea was opened, the ETT was pulled out till it was no longer visible, and the tracheostomy tube was placed into the trachea (Figure 1). The ETT position at the time of tracheostomy tube insertion during ST is also considered appropriate for PDT puncture. ST cases were collected by measuring the above ETT position in terms of the length from the tip of the tube to the median of the portal teeth.

Statistical analysis

Categorical data are expressed as numbers (percentages), and continuous variables are expressed as medians (interquartile range (IQR)) unless otherwise stated. Categorical data were compared using Pearson's chi-squared test or Fisher's direct probability test for the derivation and validation groups, and continuous variables were compared using the Student's t-test or Mann-Whitney U test. A two-sided p<0.05 value was considered statistically significant. The relationship between the measured ETT position and patient background was examined using Spearman's forward-phase relation number. A single regression analysis was performed on the parameters with the highest correlation coefficients in the derivation group to obtain a predictive model for the ETT position. The correlation between the predicted and measured values derived from the prediction equation obtained by the validation group was evaluated using Spearman's forward-phase relation number. All statistical analyses were performed using EZR (Saitama Medical Center, Jichi Medical University, Saitama, Japan), a graphical user interface for R (The R Foundation for Statistical Computing, Vienna, Austria). More precisely, it is a modified version of the R commander designed to add statistical functions frequently used in biostatistics [16].

## Results

There were 61 cases in total: 46 in the derivation group and 15 in the validation group. Table 1 summarizes the clinical characteristics and measured ETT positions of the 61 patients in the two groups. Age (p=0.519), sex (p=0.510), body height (p=0.306), length of hospital stay (p=0.487), length of ICU stay (p=0.979), SOFA score at admission (p=0.113), APACHE II score at admission (p=0.526), ETT tip position from the tracheal bifurcation (p=0.596), and ETT position before ST (p=0.368) were not significantly different between the two groups. The predominant differences between the two groups' median (IQR) or frequency (percentage) were weight (62.1 (54.2-73.2) kg vs. 51.9 (47.1-59.7) kg, p=0.008), length of intubation (6 (4-9) days vs. 10 (6.5-14.5) days, p=0.013), and in-hospital death (17 (37%) vs. 1 (6.7%), p=0.047), with a trend toward heavier weight, shorter intubation days, and higher in-hospital mortality in the derivation group and with no cases of tracheostomy complications in either group. Among the diseases causing tracheostomy, coronavirus disease 2019 (COVID-19) was more common in the derivation group (43.5% vs. 0%, p=0.001), and trauma was more common in the validation group (40% vs. 6.5%, p=0.005). Among the COVID-19 cases, there were 12 in-hospital deaths, with a median weight of 70.2 (61.1-80.8) kg and a median length of intubation of 5.5 (4-6.25) days. The median measured ETT position was 18 cm in both groups (IQR 17-19 cm in the derivation group and 16.5-18 cm in the validation group), and there was no significant difference between the two groups (p=0.163).

**Table 1:** Outcome and clinical characteristics IQR: interquartile range; ICU: intensive care unit; SOFA: Sequential Organ Failure Assessment; APACHE: Acute Physiologic Assessment and Chronic Health Evaluation; ETT: endotracheal tube; ST: surgical tracheostomy; COVID-19: coronavirus disease 2019; CPA: cardiopulmonary arrest

	Derivation group (n=46)		Validation group (n=15)		p-value
Age, median (IQR), year	69	(58.25-77.75)	76	(42-85)	0.519
Male sex, n (%)	35	(76.1)	10	(66.7)	0.510
Body height, median (IQR), cm	165.5	(160.7-170)	160	(156.5-172.5)	0.306
Weight, median (IQR), kg	62.1	(54.2-73.2)	51.9	(47.1-59.7)	0.008
Length of hospital stay, median (IQR), days	44	(29-75)	49	(37.25-62.5)	0.487
Length of ICU stay, median (IQR), days	21.5	(13-31.25)	21	(16.25-24.75)	0.979
Length of intubation, median (IQR), days	6	(4-9)	10	(6.5-14.5)	0.013
SOFA score, median (IQR)	9	(6-10)	10	(8-11.5)	0.113
APACHE II score, median (IQR)	27	(22-31)	28	(23-32)	0.526
In-hospital death, n (%)	17	(37)	1	(6.7)	0.047
ETT tip position from tracheal bifurcation, median (IQR), cm	3.9	(2.7-4.7)	3.6	(2.7-4.7)	0.596
ETT position before ST, median (IQR), cm	24	(23-25)	23	(22-26.5)	0.368
Measured ETT position, median (IQR), cm	18	(17-19)	18	(16.5-18)	0.163
Diseases leading to tracheostomy, n (%)					
COVID-19	20	(43.5)	0	(0)	0.001
Neuromuscular disease	7	(15.2)	0	(0)	0.178
CPA	4	(8.7)	3	(20)	0.348
Respiratory disease	4	(8.7)	3	(20)	0.348
Trauma	3	(6.5)	6	(40)	0.005
Airway assurance	3	(6.5)	2	(13.3)	0.589
Heart disease	3	(6.5)	0	(0)	0.569
Burn	2	(4.3)	1	(6.7)	1.000

**Figure 2:**
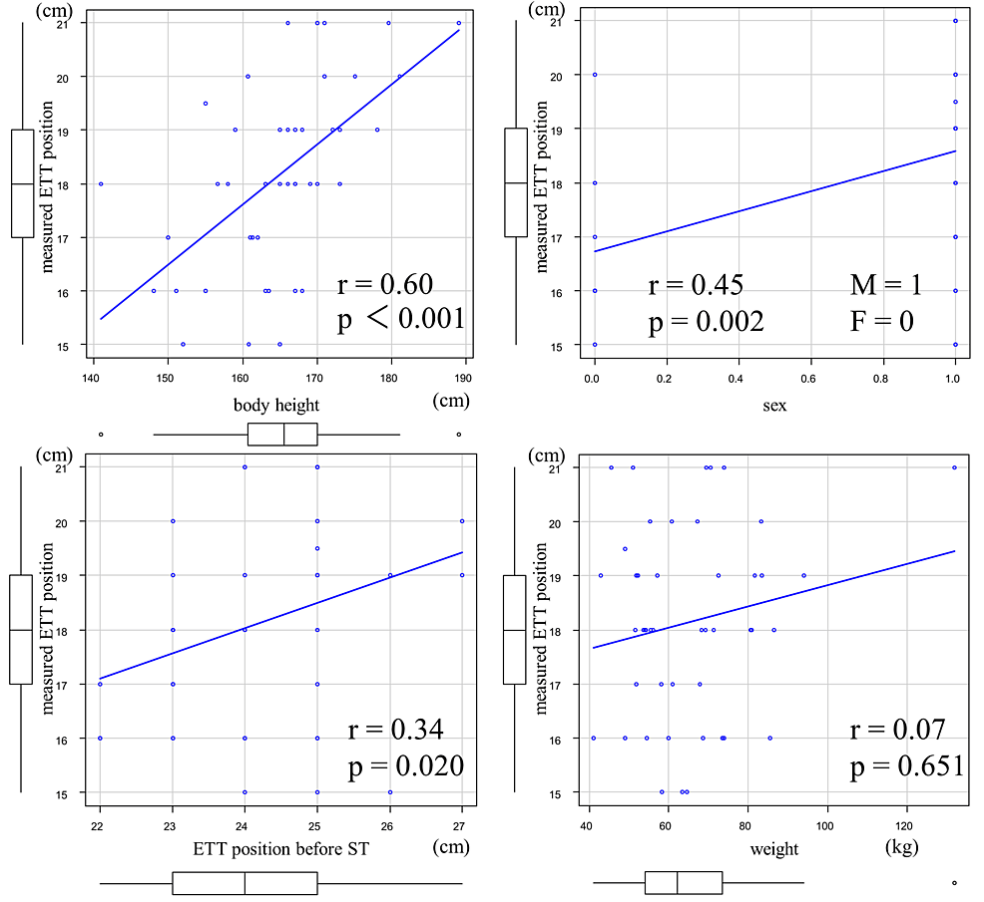
Correlation between patient characteristics and measured ETT position in the derivation group ETT: endotracheal tube; ST: surgical tracheostomy

Figure 2 summarizes the scatter plots of the measured ETT position, body height, sex, ETT position before ST, and weight in the derivation group. Correlations were found for body height (r=0.60, p<0.001) and sex (r=0.45, p=0.002), whereas ETT position before ST (r=0.34, p=0.020) showed a weak correlation. Weight showed no correlation (r=0.07, p=0.651). Single regression analysis was performed based on body height, which had the highest correlation coefficient, to derive a prediction equation for the appropriate ETT position at PDT as follows: appropriate ETT position at PDT=body height×0.112-0.323 cm (p<0.001, R-squared: 0.33, adjusted R-squared: 0.31). For example, if the patient's height were 170 cm, this prediction equation would indicate that the appropriate ETT position for PDT puncture would be 18.7 cm.

**Figure 3:**
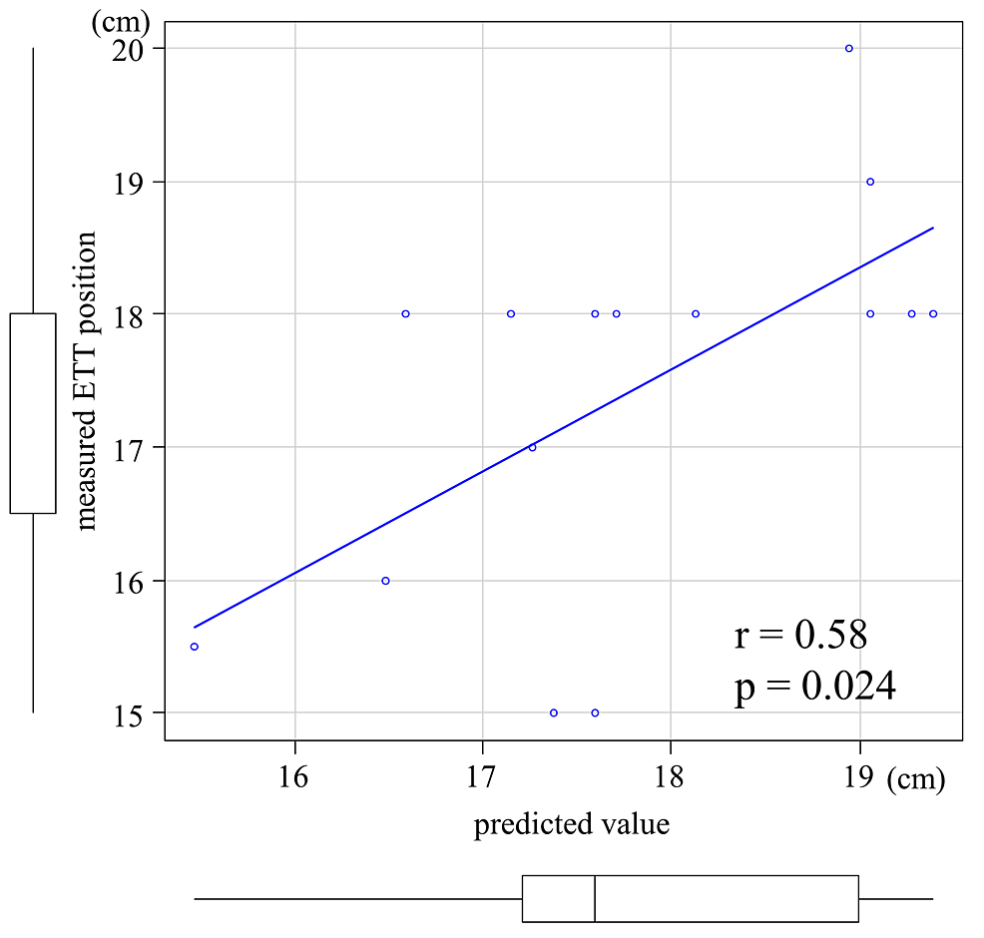
Scatter plots of the predicted and measured values in the validation group ETT: endotracheal tube

The scatter plots of the predicted and measured ETT positions in the validation group are summarized in Figure 3. The predicted and measured values correlated (r=0.58, p=0.024).

## Discussion

This study revealed the following two points: the equation "appropriate ETT position at PDT=body height×0.112-0.323 cm" was derived, and the values predicted using this equation correlated with the measured values in the validation group.

In this study, we examined the correlation between the measured ETT position and the patient's background, including sex, body height, weight, and ETT position before ST. We found that body height showed the strongest correlation (r=0.6, p<0.001). The strongest correlation between body height and the ability to predict ETT position from body height is consistent with Morgan's equation (body height/10+5 cm), which is a prediction equation for ETT position at intubation [17]. Sex also had a strong correlation (r=0.45, p=0.002) in this study; however, sex is a confounding factor for body height, which may have given rise to the correlation [18]. Conversely, the ETT position before ST was only weakly correlated (r=0.34, p=0.020). In one study, 12% of chest radiographs taken in the ICU showed abnormal ETT position [19]. In this study as well, after reviewing the chest radiographs of each case, the length of the ETT tip position from the tracheal bifurcation varied from case to case (IQR, 2.7-4.7 cm), suggesting that the inconsistent ETT position resulted in a weak correlation.

**Figure 4:**
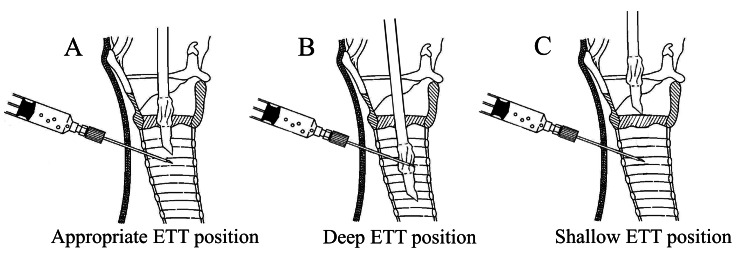
ETT position at puncture in PDT A: Appropriate ETT position. B: Deep ETT position. C: Shallow ETT position. From the Ciaglia Blue Rhino IFU images, Cook Medical. Copyright © Cook Medical. Reprinted and partially modified with permission from Cook Medical. ETT: endotracheal tube; PDT: percutaneous dilatational tracheostomy

The appropriate ETT position during PDT ranges from just above the puncture needle (Figure 4A) to the position where the cuff, which prevents accidental extubation, rests on the vocal cords (Figure 4C). This study measured the ETT position directly above the puncture needle, and the formula "body height×0.112-0.323 cm" was derived.

While comparing the two groups, the weight was heavier (p=0.008), the length of intubation was shorter (p=0.013), and in-hospital mortality was higher in the derivation group (p=0.047) compared to the validation group. The collection period for the derivation group was August 2021 to May 2022 during the COVID-19 pandemic and thus showed a higher rate of COVID-19 as the disease requiring tracheostomy (p=0.001). Obesity is a risk factor for severe COVID-19 [20,21], and the disease has a high mortality rate. The mortality rate for patients intubated with COVID-19 has been reported to be 47.3% [22]. In this study, the median weight of patients with COVID-19 was 70.2 (61.1-80.8) kg, and 12 of the 17 in-hospital deaths in the derivation group were due to COVID-19 (70.6%). When COVID-19 became severe enough to require intubation, early extubation was judged to be difficult, and tracheostomy was performed early in our hospital to promote rehabilitation (median length of intubation in COVID-19 cases: 5.5 (4-6.25) days). The COVID-19 pandemic may have caused the difference in predominance between the two groups.

The correlation coefficient between the predicted and measured values in the validation group was r=0.58, close to the correlation coefficient between the body height and measured ETT position in the derivation group (r=0.60). Although there were differences between the derivation and validation groups in terms of the length of intubation, in-hospital death, and tracheostomy-related diseases, there was no difference in the accuracy of the prediction equation between the two groups. The prediction equation presented in this study predicts an appropriate ETT position with similar accuracy, even when there are differences in patient groups, demonstrating the generality of this prediction equation. Predicting the appropriate ETT position based on body height before performing PDT could prevent accidental puncture of the ETT and accidental extubation, which may be useful for safe and short-term PDT. However, this study did not examine the extent to which PDT complications could be reduced or the time saved using this prediction equation, warranting further studies.

Limitations

This study had several limitations. First, this was a retrospective single-center observational study, which limited the external validity of the findings because of the possibility of selection bias. Second, whether the appropriate ETT position is the same for PDT and ST is unclear. Although this study was conducted assuming that the appropriate ETT position is the same for PDT and ST, it is uncertain whether this assumption is correct. Third, this study examined the ETT position directly above the puncture needle (Figure 4A) but did not examine the position of the ETT cuff over the vocal cords (Figure 4C), which poses a risk of accidental extubation. Although there may be a range of appropriate ETT positions, the present study did not examine this range.

## Conclusions

This study aimed to investigate the appropriate ETT position for PDT using ST. Our findings showed that the appropriate ETT position for PDT correlates with body height and sex. Single regression analysis was performed based on body height, which had the highest correlation coefficient, and the equation "body height×0.112-0.323 cm" was derived. The values predicted using this equation correlated with the measured values in the validation group. This predictive equation may be useful as a guide to the appropriate ETT position during PDT puncture.

## References

[1] De Leyn P, Bedert L, Delcroix M (2007). Tracheotomy: clinical review and guidelines. Eur J Cardiothorac Surg.

[2] Kollef MH, Ahrens TS, Shannon W (1999). Clinical predictors and outcomes for patients requiring tracheostomy in the intensive care unit. Crit Care Med.

[3] Freeman BD, Isabella K, Lin N, Buchman TG (2000). A meta-analysis of prospective trials comparing percutaneous and surgical tracheostomy in critically ill patients. Chest.

[4] Jackson C (1909). Tracheotomy. Laryngoscope.

[5] Veenith T, Ganeshamoorthy S, Standley T, Carter J, Young P (2008). Intensive care unit tracheostomy: a snapshot of UK practice. Int Arch Med.

[6] Vargas M, Sutherasan Y, Antonelli M (2015). Tracheostomy procedures in the intensive care unit: an international survey. Crit Care.

[7] Blot F, Melot C (2005). Indications, timing, and techniques of tracheostomy in 152 French ICUs. Chest.

[8] Kluge S, Baumann HJ, Maier C, Klose H, Meyer A, Nierhaus A, Kreymann G (2008). Tracheostomy in the intensive care unit: a nationwide survey. Anesth Analg.

[9] Krishnan K, Elliot SC, Mallick A (2005). The current practice of tracheostomy in the United Kingdom: a postal survey. Anaesthesia.

[10] Iftikhar IH, Teng S, Schimmel M, Duran C, Sardi A, Islam S (2019). A network comparative meta-analysis of percutaneous dilatational tracheostomies using anatomic landmarks, bronchoscopic, and ultrasound guidance versus open surgical tracheostomy. Lung.

[11] Klotz R, Probst P, Deininger M (2018). Percutaneous versus surgical strategy for tracheostomy: a systematic review and meta-analysis of perioperative and postoperative complications. Langenbecks Arch Surg.

[12] Beshay BN, Elbardan IM, Moustafa MA, Shehab AS (2023). A novel technique for safe blind percutaneous tracheotomy: retrospective case-series study on three hundred eighty-six patients. Ain-Shams J Anesthesiol.

[13] Oberwalder M, Weis H, Nehoda H (2004). Videobronchoscopic guidance makes percutaneous dilational tracheostomy safer. Surg Endosc.

[14] Nates JL, Cooper DJ, Myles PS, Scheinkestel CD, Tuxen DV (2000). Percutaneous tracheostomy in critically ill patients: a prospective, randomized comparison of two techniques. Crit Care Med.

[15] Mani N, Bijoor P, Cook G, Loughran S (2016). Hybrid tracheostomy. Ann R Coll Surg Engl.

[16] Kanda Y (2013). Investigation of the freely available easy-to-use software 'EZR' for medical statistics. Bone Marrow Transplant.

[17] Techanivate A, Rodanant O, Charoenraj P, Kumwilaisak K (2005). Depth of endotracheal tubes in Thai adult patients. J Med Assoc Thai.

[18] Lee BJ, Yi JW, Chung JY, Kim DO, Kang JM (2009). Bedside prediction of airway length in adults and children. Anesthesiology.

[19] Henschke CI, Pasternack GS, Schroeder S, Hart KK, Herman PG (1983). Bedside chest radiography: diagnostic efficacy. Radiology.

[20] Terada M, Ohtsu H, Saito S (2021). Risk factors for severity on admission and the disease progression during hospitalisation in a large cohort of patients with COVID-19 in Japan. BMJ Open.

[21] Kompaniyets L, Pennington AF, Goodman AB (2021). Underlying medical conditions and severe illness among 540,667 adults hospitalized with COVID-19, March 2020-March 2021. Prev Chronic Dis.

[22] Lee HJ, Kim J, Choi M, Choi WI, Joh J, Park J, Kim J (2022). Early intubation and clinical outcomes in patients with severe COVID-19: a systematic review and meta-analysis. Eur J Med Res.

